# Leucine Supplementation Decreases *HDAC4* Expression and Nuclear Localization in Skeletal Muscle Fiber of Rats Submitted to Hindlimb Immobilization

**DOI:** 10.3390/cells9122582

**Published:** 2020-12-02

**Authors:** Paula K. N. Alves, André Cruz, William J. Silva, Siegfried Labeit, Anselmo S. Moriscot

**Affiliations:** 1 Department of Anatomy, Institute of Biomedical Sciences, University of Sao Paulo, Sao Paulo 05508000, Brazil; paulaketilly@usp.br (P.K.N.A.); andrecruz@usp.br (A.C.); williamsilvaj@gmail.com (W.J.S.); 2 Faculty for Clinical Medicine Mannheim of the University of Heidelberg, Institute for Integrative Pathophysiology, Universitätsmedizin Mannheim, 68169 Mannheim, Germany; labeit@medma.de; 3 Myomedix GmbH, 69151 Neckargemund, Germany

**Keywords:** atrophy, skeletal muscle, *HDAC4*, *MAFbx*, *MYOG*, leucine supplementation

## Abstract

In this study we surveyed a rat skeletal muscle RNA-Seq for genes that are induced by hindlimb immobilization and, in turn, become attenuated by leucine supplementation. This approach, in search of leucine-atrophy protection mediating genes, identified histone deacetylase 4 (*HDAC4*) as highly responsive to both hindlimb immobilization and leucine supplementation. We then examined the impact of leucine on *HDAC4* expression, tissue localization, and target genes. A total of 76 male Wistar rats (~280 g) were submitted to hindlimb immobilization and/or leucine supplementation for 3, 7 and 12 days. These animals were euthanized, and soleus muscle was removed for further analysis. RNA-Seq analysis of hindlimb immobilized rats indicated a sharp induction (log2 = 3.4) of *HDAC4* expression which was attenuated by leucine supplementation (~50%). Real-time PCR and protein expression analysis by Western blot confirmed increased *HDAC4* mRNA after 7 days of hindlimb immobilization and mitigation of induction by leucine supplementation. Regarding the *HDAC4* localization, the proportion of positive nuclei was higher in the immobilized group and decreased after leucine supplementation. Also, we found a marked decrease of myogenin and *MAFbx*-*atrogin-1* mRNA levels upon leucine supplementation, while *CAMKII* and *DACH2* mRNA levels were increased by leucine supplementation. Our data suggest that *HDAC4* inhibition might be involved in the anti-atrophic effects of leucine.

## 1. Introduction

Skeletal muscle shows a high degree of adaptability to external pressure, such as injury and mechanical load. When the mechanical load decreases, reducing skeletal muscle burden, molecular mechanisms are triggered promoting muscle atrophy. Most of this signaling acts on protein turnover, modifying the protein synthesis and degradation balance, favoring a scenario where protein breakdown exceeds the synthesis of new proteins [1,2].

Many conditions can induce skeletal muscle atrophy, such as extended periods of limb disuse/immobilization [3,4,5], long-lasting bed rest [6], microgravity [5], physical inactivity [7], denervation [8], malignant cachexia [9], long-term corticoid treatment [10], and inflammation [11]. Generally, the atrophic process is highly integrated, embracing gene transcription and enzyme activity changes, notably, each model seems to induce skeletal muscle wasting through common and distinct changes to mass control pathways.

In disuse-induced skeletal muscle atrophy, activation of the proteolytic system named ubiquitin–proteasome is especially important. It is well established that this system is the most actively responsible for the bulk proteolysis of myofibrillar proteins in skeletal muscle [12]. The system starts by adding a polypeptide named ubiquitin (Ub) to target proteins, ultimately leading to their degradation by the proteasome. The target recognition and ubiquitin binding relies on the activity of three enzyme families: E1 ligases, which activate Ub; E2 ligases, also known as a Ub carrier enzyme; and E3 ligases, which are able to bind Ub to the target protein [2,12,13,14].

Amid E1, E2, and E3, the latter define the rate limiting step, are expressed tissue-specifically and transfer Ub to their respective target proteins, thereby triggering their degradation by the proteasome [13]. Among the different known E3 ligases in skeletal muscle, *atrogin-1* (also named *MAFbx*) and muscle ring finger 1 (MuRF1) are enriched in striated muscle and broadly known as crucial in the “atrophic program” that anticipate extensive protein breakdown. It is worth noting that these enzymes are solely expressed in skeletal and cardiac muscles [2,15,16,17].

Skeletal muscle atrophic signaling impacts multiple integrated networks. Therefore, molecules that exert broad effects including protein turnover regulation might be suitable to combat muscle wasting. In this regard, leucine has been described as a nutritional intervention that augments protein synthesis while minimizing skeletal muscle protein degradation [18]. Several studies showed that leucine supplementation can attenuate mass loss driven by hindlimb immobilization in rat soleus muscle [4,19,20,21].

Here, we prospected unanticipated pathways modulated by leucine supplementation in a model of disuse by hindlimb immobilization. By performing an RNA-Seq we noticed that histone deacetylase 4 (*HDAC4*) could be highly upregulated by hindlimb immobilization, generating the hypothesis that leucine can mitigate the rise in *HDCA4* levels in the skeletal muscle during hindlimb immobilization.

*HDAC4* has been previously reported as an important factor in skeletal muscle mass loss. Denervation strongly upregulates *HDAC4* gene and protein levels driving *HDAC4* nuclear translocation, leading to impaired dachshund homolog 2 (*DACH2*) expression and resulting in myogenin upregulation [22,23]. The importance of the *HDAC4*/myogenin axis was confirmed mechanistically by *HDAC4* knockdown and overexpression experiments [22,23,24]. Interestingly, these authors showed that myogenin can increase the activity of *HDAC4* promoter, suggesting a myogenin positive feedback effect on *HDAC4* [23]. Furthermore, myogenin can activate the promoter of both *atrogin-1* and MuRF1, establishing a link between the *HDAC4*/*DACH2*/myogenin circuit and ubiquitin-dependent proteolysis in skeletal muscle [25]. Finally, consistent with a role of *HDAC4* in atrophy signaling, its expression is increased in several atrophy models, such as the spinal muscular atrophy model [26], aging [27], and in hindlimb immobilization [3].

In the present study we identified *HDAC4* in rat skeletal muscles as responding both to hindlimb immobilization and to leucine supplementation. Our data suggest that the *HDAC4*–leucine signaling axis might represent an important pathway in the context of skeletal muscle atrophy.

## 2. Materials and Methods

### 2.1. Experimental Animals

In total, 76 male Wistar rats (±280 g) were used in this study. All animals were maintained in a bioterium with controlled temperature (24 ± 1 °C, 12-h light–dark cycle) and standard food (Nuvilab CR-1, Nuvital-Quimtia, Brazil) and water were offered *ad libitum*. The animals were randomly divided into four category groups: the control groups (3 days *n* = 6, 7 days *n* = 9 and 12 days *n* = 4) received only saline via gavage for 3, 7 and 12 days; the immobilized groups (Imm 3 d *n* = 6, Imm 7 d *n* = 9 and Imm 12 d *n* = 4) also received saline via gavage for 3, 7 and 12 days and the left posterior paw was immobilized to full plantar flexion position; the leucine groups (Leu 3 d *n* = 6, Leu 7 d *n* = 9 and Leu 12 d *n* = 4) received leucine diluted in saline via gavage for 3, 7 and 12 days; and the immobilized + leucine groups (Imm 3 d + Leu *n* = 6, Imm 7 d + Leu *n* = 9 and Imm 12 d + Leu *n* = 4) received leucine diluted in saline via gavage for 3, 7 and 12 days and the left posterior paw was immobilized. Leucine (L-Leucine, Sigma—#L8000, St. Louis, MO, USA) was orally administered once a day at a dose of 1.35 g/kg body mass (modified from [4]), starting 3 days prior to immobilization.

This study was approved by the University of São Paulo (Institute of Biomedical Sciences) ethics committee under the protocol #2034200718 (registered on 26 July 2018) and followed the Code of Practice for the Housing and Care of Animals Used in Scientific Procedures.

### 2.2. Hindlimb Immobilization

Rats submitted to immobilization and/or leucine supplementation were anesthetized by an intraperitoneal injection of a ketamine and xylazine solution (100 and 10 mg/kg, respectively) and then prepared for immobilization procedures. The left hindlimb was fixed in full plantar flexion position with plaster and then covered in a steel net. The animals were monitored daily for controlling plaster chewing, scratches, and motility problems. After 3, 7 or 12 days, the rats were fasted for four hours before euthanasia, in order to ensure that all animals were in the same metabolic state. The animals were euthanized by cervical dislocation, and the soleus muscle was quickly harvested, weighed, and transversally cut into two parts: one half was immersed in hyper-cooled isopentane for 60 s and then snap-frozen in liquid nitrogen for tissue analysis, and the other half was snap-frozen in liquid nitrogen to be used in gene and protein expression analysis. For storage, the samples were kept at −80 °C.

### 2.3. RNA-Seq Analysis

Total RNA was isolated from the soleus muscle by homogenization in TRIzol reagent following the manufacturer’s protocol. RNA integrity was verified using a Nano BioAnalyzer 2100 (Agilent, Santa Clara, CA, USA), and samples with an RIN (RNA Integrity Number) greater than 7 progressed through sequencing. mRNA library and sequencing were carried out at the Genomic Center of Escola Superior de Agricultura “Luiz de Queiroz” (ESALQ-USP, Piracicaba, Brazil), by TruSeq Stranded mRNA Sample LS protocol (Illumina, San Diego, CA, USA) and HiSeq 2500 SBS High Output protocol (2 × 100 pb) (Illumina) sequencing platform, respectively. The reads had adaptor sequences, and low-quality bases were removed by trimmomatic software (v. 0.36). The survivor sequences (quality scores > 20) were used in further analysis. The reads were mapped to the rat genome (Rnor 6.0) using HISAT2 software [28,29,30], and hits were summarized for genes using the featureCounts tool [31]. Differential expression analysis was performed through the edgeR package [32,33]; in short, genes with low counts were filtered from the libraries (<15) and survivors were normalized by library size and biological variation. For selecting differentially expressed genes, first, the “exact test” was employed, and subsequently, for stringent analysis we used a cut-off log2 > 2 for up- or downregulated genes. Furthermore, p and false discovery rate (FDR) values < 0.05 were considered for differentially expressed genes.

### 2.4. Cross-Sectional Area (CSA) Analysis

Muscles were transversely sectioned (10 µm thick) using a cryostat (Leica CM1850 UV, Wetzlar, Germany) and then stained with hematoxylin and eosin. Photomicrographs were obtained by Axio Scope.A1 microscopy (Carl Zeiss Microscopy GmbH, Göttingen, Germany). The CSA of muscle fibers was obtained on the software ImageJ (v. 1.45s, National Institutes of Health, Bethesda, MD, USA), and approximately 800–1200 fibers per group were analyzed.

### 2.5. Dry Weight

The dry weight protocol was based on Gissel [34]. In brief, after removal, the soleus was allowed to dry in an incubator at 60 °C and the weight monitored daily. The muscles continued to be weighed at the same time of day until the weight became stable.

### 2.6. Immunofluorescence

Muscle cross-sections were fixed at room temperature in 4% PFA for 10 min, washed (three times for 5 min) with TBS-T (tris-buffered saline, 0.5 M NaCl, 50 mM tris-HCl pH 7.4 with 0.3% TRITON X-100). Subsequently, the sections were incubated with blocking solution (1 h, 1% bovine serum albumin in TBS-T) and then incubated overnight at 4 °C with primary antibody rabbit anti-*HDAC4* (1:250; Cell Signaling, #7628). After primary antibody incubation, the slides were washed with TBS-T 0.3% (three times for 5 min) and submitted to the secondary antibody (1:250 Cy3 Donkey Anti-Rabbit, Jackson ImmunoResearch, West Grove, PA, USA) in blocking solution (1 h) and washed (three times for 5 min in TBS-T). The slides were then mounted with coverslips using mounting media containing 4′,6-diamidino-2-phenylindole (DAPI) (cat# H-1200, Vectashield, Vector Labs, Burlingame, CA, USA). Digital acquisitions and the nuclear colocalization analysis were performed using confocal microcopy equipment (Zeiss LSM 780-NLO), which uses excitation laser rays that allow the acquisition of images of two proteins along the deep Z axis, capturing proteins that are within the nucleus in their totality. The nuclei were selected manually, and through the equipment’s software the percentage of *HDAC4* nuclear colocalization with DAPI was determined.

### 2.7. Western Blotting

The tissue samples were grinded using a liquid-nitrogen-chilled mortar. The generated powder was homogenized in RIPA buffer (1 mM EDTA, pH 7.4, 0.0625% sodium deoxycholate, 0.0625% nonidet P-40, 6.2 mM sodium phosphate, and protease and phosphatase inhibitor cocktail—Thermo Scientific cat#78445). Homogenates were maintained immersed in ice (30 min), then centrifuged (10,000 rpm for 10 min at 4 °C), finally the supernatant was quantified (Bradford method).

Isolated total protein (25 µg per lane) was loaded in a 12% polyacrylamide gel (SDS-PAGE) and submitted to electrophoresis (60–120 V for 60–120 min). Proteins were transferred to a polyvinylidene difluoride membrane of 0.45 µm (Thermo Fisher Scientific, CAT: 88518, Rockford, IL, USA) in a semi-dry system (20 V for 120 min). To verify a homogeneous loading, membranes were stained with Ponceau S. Next, membranes were blocked with 5% BSA in tris-buffered saline with Tween (0.5 M NaCl, 50 mM tris-HCl pH 7.4, 0.1% Tween 20) for 1 h. Subsequently, membranes were washed (three times for 5 min) with tris-buffered saline containing 0.1% Tween, followed by overnight incubation at 4 °C with primary antibody. Primary antibodies were as follows: rabbit anti-*HDAC4* (1:1000; Cell Signaling, #7628) and rabbit anti-GAPDH (1:3000; Cell Signaling, #2118). Then, membranes were incubated with a secondary antibody (1:30,000, goat anti-rabbit peroxidase cat#111035003, Jackson ImmunoResearch) in a solution containing 5% BSA in tris-buffered saline and Tween 0.1% (1 h at room temperature). Then, the membranes were washed (three times for 5 min, tris-buffered saline and Tween). Luminata^TM^ (cat#WBLUF0500, Millipore, Burlington, MA, USA) reagent was used to visualize specific bands, which were analyzed in the Fusion FX5 XT (Vilber Lourmat, Collégien, France) imaging system. Variations in loading were monitored by GAPDH.

### 2.8. RNA Extraction and Real-Time PCR

Muscle samples (±20 mg) were homogenized by Polytron©, and Trizol was used to isolate total RNA (Invitrogen©, Carlsbad, CA, USA) following the manufacturer’s recommendations. The pellets were resuspended in ultrapure water, and RNA concentrations were obtained at 260 nm absorbance (Eppendorf©, Wilmington, DE, USA). The 260/280 ratio was determined and used as a reference for RNA purity, and RNA integrity was verified on an agarose denaturing gel stained with ethidium bromide.

For gene expression analysis, cDNA synthesis was performed with 2 µg of total RNA, and 5% of this reaction was used to perform the real-time PCR (with EvaGreen qPCR Supermix, Solis Biodyne©, Tartu, EST, 200 nM of each primer, Table S1). PCR was run at 95 °C for 12 min, followed by 40 cycles of 95 °C for 15 s, 60 °C for 30 s, and 72 °C for 30 s. *Cyclophilin A* RNA was used as housekeeping gene. For further details see reference [4].

### 2.9. Statistical Analysis

Multiple comparisons were established using either one-way ANOVA followed by Tukey’s posthoc test (for parametric data) or Kruskal-Wallis test of one-way ANOVA followed by Dunn’s posthoc test (for non-parametric data) [35]. Data were presented as mean ± SD or SEM. GraphPad Prism 7.0 was used for analysis and *p* < 0.05 was considered significant.

## 3. Results

### 3.1. Biometric Measurements

First, we verified the impact of immobilization and leucine supplementation on soleus muscle dry mass adjusted by tibia length. Following 3 and 7 days of hindlimb immobilization, dry mass of soleus decreased by 23% (Control: 7.69 ± 0.69 mg/cm vs. Imm 3 d: 6.58 ± 0.91 mg/cm) and 44% (Control: 6.11 ± 0.1 mg/cm vs. Imm 7 d: 3.42 ± 0.1 mg/cm), respectively, and leucine supplementation attenuated this loss at 7 days by 28% (Control: 6.11 ± 0.1 mg/cm vs. Imm 7 d + Leu 4.38 ± 0.2 mg/cm). Accordingly, whole muscle cross-sectional area also decreased by 45% (Control: 9.57 ± 0.28 mm^2^ vs. Imm 7 d: 5.33 ± 0.52 mm^2^) by hindlimb immobilization after 7 days, an effect that was also attenuated by leucine supplementation by 19% (Control: 9.57 ± 0.28 mm^2^ vs. Imm 7 d +Leu: 7.75 ± 1.30 mm^2^). At days 3 and 7 we detected neither a significant decrease in whole muscle cross-sectional area nor a change in soleus dry weight by leucine alone (Table 1). Body mass was not altered among the groups (Table 1).

### 3.2. Hindlimb Immobilization Induced Skeletal Muscle Atrophy, Which Was Attenuated by Leucine Supplementation

**Figure 1:**
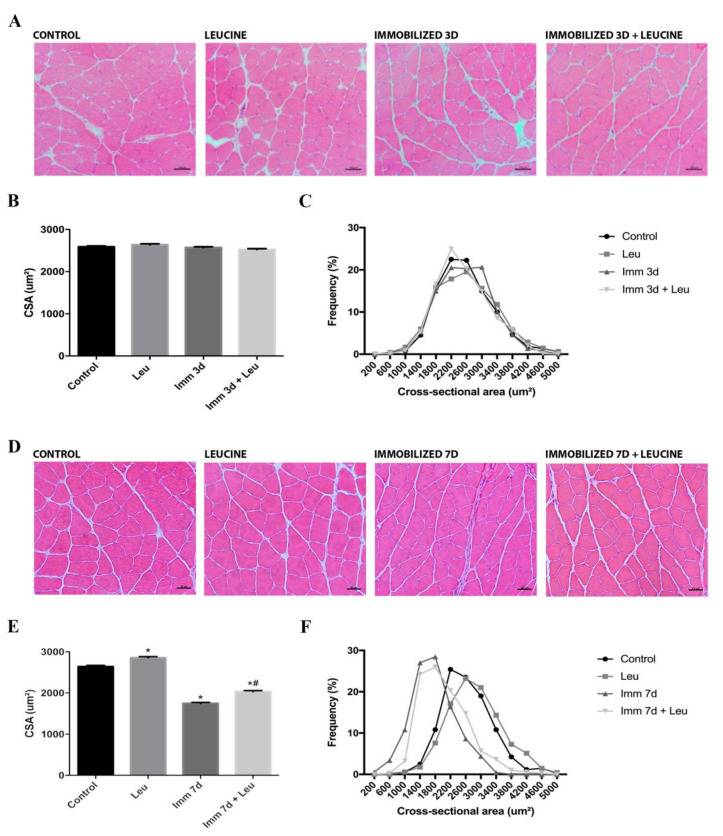
Morphometric analysis after 3 and 7 days of immobilization and leucine supplementation. (**A**) Representative hematoxylin–eosin staining (HE) photomicrographs in soleus muscle after 3 days of immobilization (*n* = 4, scale bar 50 µm). (**B**) Soleus muscle average fiber cross-sectional area (CSA) and (**C**) distribution from control, leucine supplemented, immobilized, and immobilized supplemented with leucine after 3 days (*n* = 4). (**D**) Representative HE photomicrographs in soleus muscle after 7 days of immobilization (*n* = 5, scale bar 50 µm). (**E**) Soleus muscle average fiber CSA and (**F**) distribution from control, leucine supplemented, immobilized, and immobilized supplemented with leucine after 7 days (*n* = 5). Data were expressed as mean ± SD. Statistical analysis included Kruskal-Wallis test of one-way ANOVA followed by Dunn’s posthoc test. Two independent experiments were performed. * *p* < 0.05 vs. Control group; ^#^ *p* < 0.05 vs. Immobilized 7 d group.

We also measured fiber CSA and detected, as expected, that 7 days of hindlimb immobilization caused a decrease of 34% (Control: 2645 ± 25.53 µm^2^ vs. Imm 7 d: 1749 ± 21.45 µm^2^) in this parameter, although we did not detect a significant drop after 3 days of hindlimb immobilization. Leucine supplementation, as expected, was able to attenuate the decrease in CSA induced by hindlimb immobilization after 7 days by 23% (Control: 2645 ± 25.53 µm^2^ vs. Imm 7 d + Leu: 2035 ± 23.92 µm^2^). Moreover, leucine alone induced an 8% (Control: 2645 ± 25.53 µm^2^ vs. Leu 7 d: 2856 ± 26.83 µm^2^) increase in CSA (Figure 1). Overall, these results confirm that both hindlimb immobilization and leucine supplementation were successfully established.

### 3.3. RNA-Seq Analysis and HDAC4 mRNA Expression Validation for 3 and 7 Days of Hindlimb Immobilization

**Figure 2:**
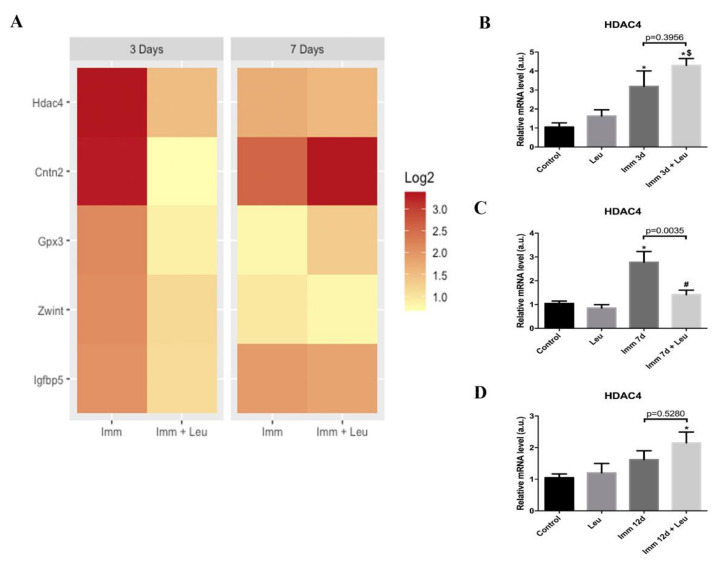
Genes responsive to hindlimb immobilization downregulated by leucine supplementation and *HDAC4* validation at 3, 7 and 12 days. (**A**) Heat maps showing top differentially expressed genes strongly upregulated at 3 days of hindlimb immobilization that were softened by leucine supplementation; the 7-day timepoint is shown as time reference (*n* = 2 per group). (**B**) *HDAC4* mRNA levels were determined by real-time PCR at 3 days (*n* = 6), (**C**) 7 days (*n* = 8) (**D**) and 12 days (*n* = 4) after immobilization (two independent experiments were performed for 3 and 7 days, and one independent experiment was performed for 12 days). *Cyclophilin A* expression was used as housekeeping gene. Data are expressed as mean ± SEM. Statistical analysis included one-way ANOVA followed by Tukey’s posthoc test. * *p* < 0.05 vs. control group; ^#^ *p* < 0.05 vs. immobilized 7 d group; ^$^ *p* < 0.05 vs. leucine group.

To find strong targets of leucine action, we first selected the genes highly upregulated by 3 days after immobilization (log2 ≥ 2). The complete set of regulated genes is presented in Supplementary Table S2, and we have detected 77 genes that were highly upregulated. We also highlight the top 20 regulated genes in Supplementary Table S3. Out of those genes, we selected the ones that were not differentially expressed in the hindlimb the immobilization + leucine group, as compared to the control group. By using this rigorous criterion we selected only five genes (Figure 2A). Those genes were plotted in a heat map paired with 7-day data to visualize the stability of the response. *HDAC4* caught our attention due to its strong response to hindlimb immobilization and the attenuating effect of leucine supplementation (Figure 2A).

Next, we evaluated *HDAC4* gene expression at 3, 7 and 12 days using real-time PCR. We observed that *HDAC4* mRNA levels increased after 3 and 7 days of hindlimb immobilization (~3 fold for both timepoints), validating our RNA-Seq data. Notably, leucine supplementation was able to attenuate the immobilization induction of *HDAC4* only in the 7-day group (Figure 2C). To determine whether at longer time periods leucine would retain the attenuative effect on *HDAC4*, we evaluated mRNA levels at 12 days of hindlimb immobilization. The results showed that hindlimb immobilization alone did not change mRNA *HDAC4* expression at 12 days (Figure 2D). On the other hand, in leucine supplementation group with hindlimb immobilization we observed increased *HDAC4* mRNA levels (~2 fold) (Figure 2D).

### 3.4. Leucine Downregulates HDAC4 Protein Expression at 7 Days of Hindlimb Immobilization

**Figure 3:**
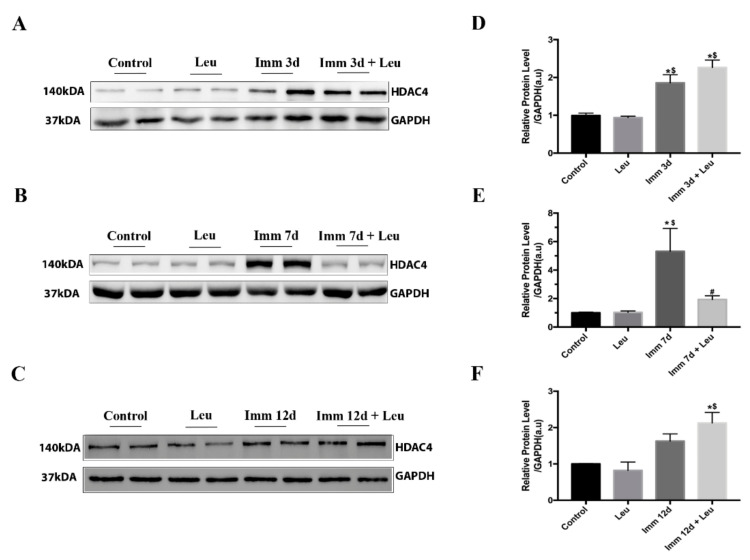
Effect of leucine on HDAC4 protein expression. Protein levels of HDAC4 were determined at 3 days, 7 days and 12 days of immobilization in leucine supplemented, immobilized, and immobilized leucine supplemented groups. (**A**) HDAC4 Western blot showing representative bands at 3 days, (**B**) 7 days, and (**C**) 12 days. Note that Control and Leucine groups were submitted to different conditions; 3, 7 and 12 days of saline and leucine supplementation respectively. (**D**) Densitometry analysis at 3 days (*n* = 6 per group), (**E**) 7 days (*n* = 9 per group), and (**F**) 12 days (*n* = 4 per group). Two independent experiments were performed for 3 and 7 days, and one independent experiment was performed for 12 days. GAPDH protein level was used as loading control. Data are expressed in arbitrary units (a.u.) as mean ± SEM. Statistical analysis included one-way ANOVA followed by Tukey’s posthoc test. * *p* < 0.05 vs. control group; ^#^ *p* < 0.05 vs. immobilized 7 d group; ^$^ *p* < 0.05 vs. leucine group.

HDAC4 protein levels were in agreement with gene expression results. Hindlimb immobilization for 3 and 7 days increased HDAC4 protein levels (~2 and 5-fold, respectively), on the other hand 12 days did not change HDAC4 protein levels. In line with real time PCR data, leucine supplementation blocked HDAC4 protein expression rise only after 7 days of hindlimb immobilization (Figure 3).

### 3.5. Leucine Reduces the Percentage of HDAC4 Positive Nuclei Accumulation Induced by Immobilization

**Figure 4:**
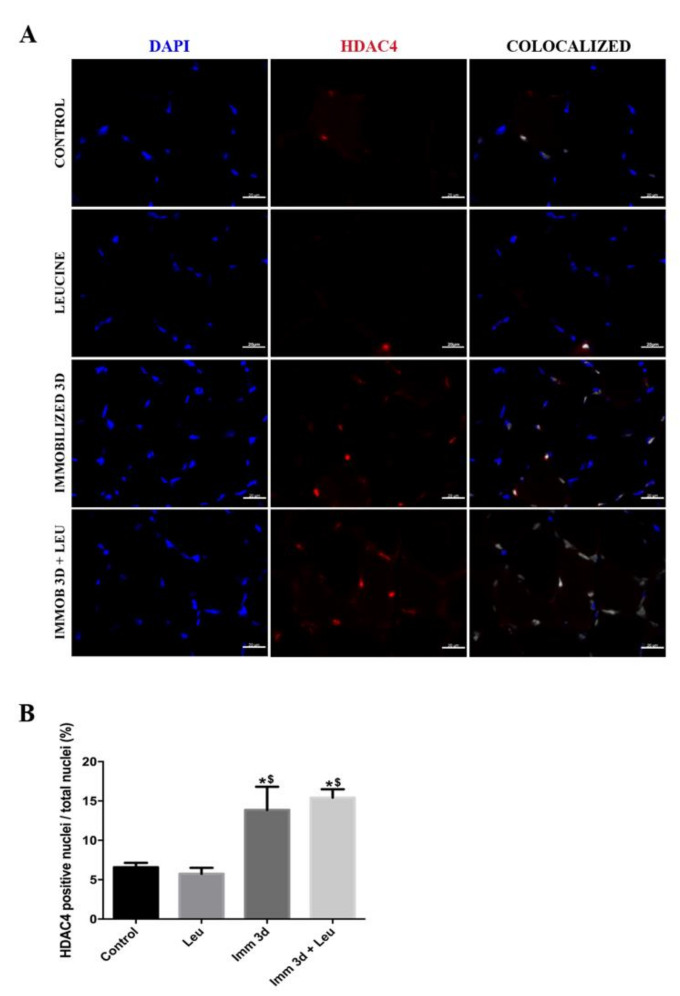
HDAC4 colocalization analysis after 3 days of immobilization and leucine supplementation. (**A**) Representative immunofluorescence photomicrographs of HDAC4 in soleus muscle after 3 days of immobilization, HDAC4 (red), DAPI (blue, used for nuclei identification), and colocalized (white) scale bar 20 µm. Less intense puncta reflect nuclei identified at either different focal planes or nuclei that were cut close at the edge. (**B**) Number of positive HDAC4 nuclei per total nuclei (%) (*n* = 3 per group, one independent experiment was performed). Data are expressed as mean ± SEM. Statistical analysis included one-way ANOVA followed by Tukey’s posthoc test. * *p* < 0.05 vs. control group; ^$^ *p* < 0.05 vs. leucine group.

**Figure 5:**
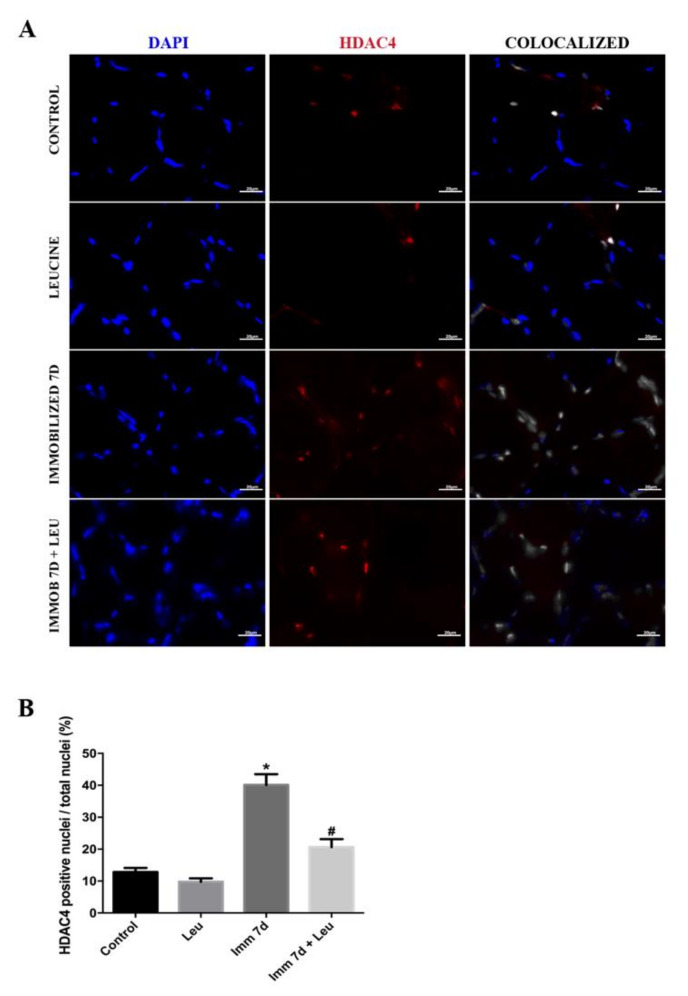
HDAC4 colocalization analysis after 7 days of immobilization and leucine supplementation. (**A**) Representative immunofluorescence photomicrographs of HDAC4 in soleus muscle after 7 days of immobilization, HDAC4 (red), DAPI (blue, used for nuclei identification), and colocalized (white) scale bar 20 µm. Less intense puncta reflect nuclei identified at either different focal planes or nuclei that were cut close at the edge. (**B**) Number of HDAC4 positive nuclei per total nuclei (%) (*n* = 5 per group, two independent experiments were performed). Data are expressed as mean ± SEM. Statistical analysis included one-way ANOVA followed by Tukey’s posthoc test. * *p* < 0.05 vs. control group; ^#^ *p* < 0.05 vs. immobilized 7 d group.

Next, we investigated HDAC4 tissue localization because its nuclear translocation is essential for triggering its target genes. By confocal immunofluorescence analysis, we observed that 3 days of hindlimb immobilization induced an increase in the number of HDAC4 positive nuclei (~2 fold), an effect that was not seen in the hindlimb immobilized and leucine group (Figure 4). At 7 days of hindlimb immobilization, the number of HDAC4 positive nuclei increased (~3 fold) and leucine supplementation vigorously attenuated this effect (~50% attenuation) (Figure 5). Since the attenuating effects of leucine supplementation on *HDAC4* mRNA and protein levels and immunolocalization were observed only after 7 days of hindlimb immobilization and leucine supplementation, we decided to focus on this timepoint for concluding analysis.

### 3.6. Impact of Leucine on Gene Expression of HDAC4 Pathway-Related Components during 7 Days of Hindlimb Immobilization

Finally, we explored molecular components directly related to the *HDAC4* atrophic effect: *CAMKII*, *MYOG*, *DACH2*, and *atrogin-1*/*MAFbx* gene expression.

**Figure 6:**
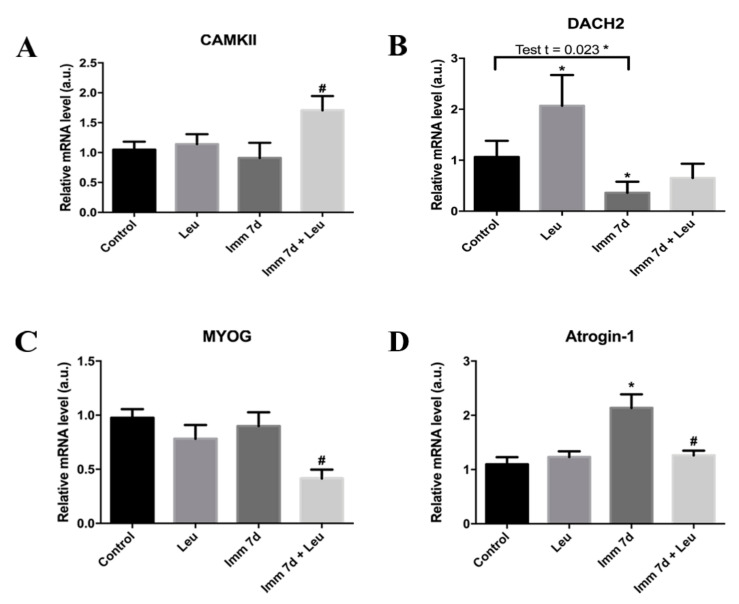
Effect of leucine on the *HDAC4* canonical pathway. *CAMKII*, *DACH2*, *MYOG*, and *atrogin-1* mRNA levels (**A**–**D**, respectively) were determined by qPCR 7 days after immobilization in leucine supplemented, immobilized, and immobilized leucine supplemented groups (*n* = 5–8 per group, two independent experiments were performed). *Cyclophilin A* expression was used as housekeeping gene. Data are expressed as mean ± SEM. Statistical analysis included one-way ANOVA followed by Tukey’s posthoc test. * *p* < 0.05 vs. control group; ^#^ *p* < 0.05 vs. immobilized 7 d group.

When 7 days had passed after immobilization, *CAMKII* mRNA levels were altered neither by leucine supplementation alone nor by hindlimb immobilization. On the other hand, leucine supplementation in hindlimb immobilized animals promoted increased *CAMKII* mRNA levels (~1.7 fold) when compared to the immobilized group (Figure 6).

We observed that *DACH2* mRNA levels were elevated by leucine supplementation alone (~2 fold), and only hindlimb immobilization caused a decrease in its mRNA levels (0.5 fold, significant in Student’s *t*-test). Leucine supplementation was not able to soften the *DACH2* downregulation induced by immobilization at this timepoint (Figure 6).

Regarding *MYOG* we noticed a decrease in mRNA levels exclusively in the immobilized and leucine supplemented group (Figure 5). Conversely, *atrogin-1*/*MAFbx* mRNA expression was increased (~2 fold) by immobilization, and leucine supplementation was able to block this effect (Figure 6).

## 4. Discussion

In the present study we identified that leucine strongly attenuates the positive response of *HDAC4* to hindlimb immobilization. Furthermore, leucine is also capable of attenuating the *HDAC4* positive nuclei accretion induced by hindlimb immobilization.

The hindlimb immobilization employed in this study caused a drop in skeletal muscle mass and myofiber cross-sectional area clearly detectable at day 7. These effects were attenuated by leucine, notably at day 7, which prove that the hindlimb immobilization model used herein was successfully employed, as compared to previous reports [4,36,37]. It is worth to stress that this protective effect occurs preferentially in slow twitch muscles, because type I fiber is the one that directly benefits from leucine anti-atrophic action [4,37].

The experiments performed herein were motivated by a RNA-Seq assay from soleus muscle of hindlimb immobilized animals supplemented with leucine (Figure 2) using as stringent identification criteria of responsiveness to hindlimb immobilization of at least 4 fold (log2 ≥ 2) and attenuation of this response by leucine by 40% in one of the timepoints addressed. Only five genes attained these criteria, including *HDAC4* as the top candidate (Figure 2). It is important to mention that our RNA-Seq assay has only two points per group; therefore, it was a rather prospective strategy, not aiming to provide a solid statistical basis. When analyzed by real-time PCR and Western blot, we confirmed the expected rise in *HDAC4* gene and protein expression by hindlimb immobilization and verified that leucine was especially efficient in attenuating this effect 7 days after the onset of the immobilization procedure. These findings confirm that prospective strategies using RNA-Seq analysis are potent tools for discovery; nonetheless, validation is essential for confirmation. It should be noted that the *HDAC4* data obtained herein are in line with the finding that the leucine anti-atrophic effect has a delay in such a way that at 3 days of hindlimb immobilization no protective effects are noted, whereas it becomes evident at 7 days [4,36,37]. Furthermore, at 12 days of hindlimb immobilization, the anti-atrophic effect of leucine is no longer detectable. Therefore, *HDAC4* response to hindlimb immobilization is transient.

Other than the identification of a connection between *HDAC4* and leucine, one of the main findings of the present study regards the possible cellular mechanisms by which leucine acts. Since *HDAC4* nuclear localization is required to induce atrophy-related gene transcription such as *atrogin-1*, we evaluated the effect of leucine on *HDAC4* nuclear localization: in muscles from hindlimb immobilized animals, about ~40% of nuclei were *HDAC4* positive, which is about four times higher than controls (Figure 5). Leucine supplementation attenuates this response by about one half, and interestingly, HDAC4 protein level is similarly attenuated by leucine (Figure 3). Of note, apparent differences in HDAC4 and DAPI intensities or areas (Figure 4A and Figure 5A) reflect signals either identified at distinct focal planes or marginally sectioned.

It is widely recognized that the mechanisms controlling nuclear protein transport are complex, and they were not addressed in the present study. Nonetheless, based on the similarities between protein levels and nuclear localization, one could envisage that the *HDAC4* nuclear localization strictly reflects the level of available protein, rather than being a mechanism involving controlled access to nuclei.

One important aspect to be considered, not experimentally addressed in the present work, regards how *HDAC4* is mechanistically modulated by leucine. The literature is rather scarce in addressing the direct molecular interactions of leucine, but it has been recently proposed that this amino acid directly interacts with Sestrin2 [38]. Sestrin has been recently described as an anti-atrophic agent which stimulates mTORC1 and consequently decreases protein degradation [39]. Furthermore, it has been shown that sustained mTORC1 activation impairs the nuclear import of *HDAC4* [40]. Another promising molecule is VPS34, a class-III PI3K that can activate MTORC1 [41]. VPS34 has been shown to be activated by leucine, which can therefore induce mTORC1 [40]. In fact, our group has shown that leucine’s ability to prevent FoxO3a nuclear localization is dependent on VPS34 [36]. VPS34 is also activated by LRS (leucyl-tRNA synthetase), the enzyme that combines leucine with the respective tRNA. It has been reported that LRS can activate VPS34 and therefore also stimulate mTORC1 [41]. Nevertheless, the exact link between leucine and the *HDAC4* axis is still not defined.

To provide further understanding of the mechanism of action of leucine, we have addressed the main components involved in *HDAC4* action: *CAMKII*, *MYOG*, *DACH2*, and *atrogin-1*/*MAFbx*. Our results indicate that *CAMKII* modulation by leucine might actually be one of the central factors in *HDAC4* molecular action. *CAMKII* mRNA levels are significantly elevated by leucine, particularly in animals submitted to hindlimb immobilization (Figure 6). *CAMKII* is a calcium-calmodulin dependent kinase that is strongly activated in muscle mechanical stress conditions, such as during exercise, and is also able to promote *HDAC4* nuclei exporting, limiting its action in modulating gene transcription [42,43,44].

According to current knowledge, *HDAC4* is able to inhibit the transcriptional factor *DACH2* [22]; therefore, under leucine, one would predict that this amino acid would exert a positive effect on *DACH2*. Herein, we showed that leucine can increase *DACH2* mRNA levels by about 2 fold in control muscles, an effect that we did not observe in muscles of animals submitted to immobilization (Figure 6). Although we did not detect increased levels of *DACH2* mRNA levels in immobilized animals, it should be taken into consideration that these samples were analyzed 7 days after, immobilization and at early timepoints *DACH2* mRNA might have been modulated by leucine. In fact, we do see a downregulation in *MYOG* mRNA levels in immobilized animals submitted to leucine supplementation, indicating that the known connection between *DACH2* and *MYOG* is in fact operating in the context of leucine [24]. If *MYOG* is downregulated by leucine, one would expect to observe a consequence in a target gene. In fact, we observed that *atrogin-1* (a target of *MYOG*) [25] mRNA levels are downregulated in immobilized animals supplemented with leucine as compared to solely immobilized ones (Figure 6). Future studies addressing this regulatory axis at the functional protein level will be useful.

According to the results herein, leucine can also act as an *HDAC4* blocker in humans, including decreased HDAC4 protein levels and decreased nuclear localization. Leucine supplementation has been intensely investigated in humans, and varying results have been reported [45,46,47,48,49]. Differences in age of subjects, time of leucine supplementation, and differences in dosage might explain the conflict between these results [45,46,47,48,49].

## 5. Conclusions

In summary, the results herein suggest that *HDAC4* inhibition might be an important way in which leucine exerts its anti-atrophic effects.

## Figures and Tables

**Table 1:** Body and muscle measurements analysis after 3 and 7 days of immobilization and leucine supplementation. Data were expressed as Mean ± SD. Statistical analysis included one-way ANOVA followed by Tukey’s posthoc (*n* = 6–7 per group, two independent experiment were performed). * *p* < 0.05 vs. Control Group; ^#^ *p* < 0.05 vs. Immobilized Group.

Biometric Feature	Control		Leucine		Immobilized		Immobilized + Leucine	
	3 Days	7 Days	3 Days	7 Days	3 Days	7 Days	3 Days	7 Days
Body Weight (g)	297.4 ± 34.7	361.2 ± 44.2	280.0 ± 20.8	342.7 ± 45.8	282.8 ± 23.3	302.9 ± 48.6	280.3 ± 31.6	312.1 ± 20.0
Soleus Dry Weight/Tibia (mg/cm)	7.69 ± 0.69	6.11 ± 0.1	6.58 ± 0.91	7.05 ± 0.7	5.91 ± 0.68 *	3.42 ± 0.1 *	6.22 ± 0.53	4.38 ± 0.2 *^,#^
Whole Area of Soleus Muscle (mm^2^)	8.50 ± 1.49	9.57 ± 0.28	8.53 ± 1.09	8.45 ± 0.98	7.20 ± 1.68	5.33 ± 0.52 *	7.83 ± 0.87	7.75 ± 1.30 ^#^

## Supplementary Materials

The following are available online at https://www.mdpi.com/2073-4409/9/12/2582/s1, Table S1: List of genes and primers used for gene expression analysis; Table S2: Differential expressed (DE) genes from rat soleus muscle compared with control group; Table S3: Top 20 Differential expressed (DE) genes up-regulated and downregulated within soleus muscle from rats when compared to control group. Symbol: Unique abreviation from a gene. logFC: Logarith of fold change of gene expression. logCPM: Logarith of counts per million. FDR: False discovery rate; Table S4: Differential expressed (DE) genes up-regulated and down-regulated within soleus muscle from rats when compared to control group.
