# Supplementary material for: Leucine Supplementation Decreases HDAC4 Expression and Nuclear Localization in Skeletal Muscle Fiber of Rats Submitted to Hindlimb Immobilization
Source: Cells. 2020 Dec 2;9(12):2582. doi: 10.3390/cells9122582 (PMC7761616; doi:10.3390/cells9122582)
Supplement: Supplementary file 1 [file cells-09-02582-s001.zip › cells-958374 supp conv/Supplemental Table 1.pdf]

Supplemental Table 1: List of genes and primers used for gene expression analysis.			
**GENE**	**GENE ID**	**Forward 5′-3′**	**Reverse 3′-5′**
Atrogin-1	NM_133521.1	GAA CAG CAA AAC CAA AAC TCA GTA	GCT CCT TAG TAC TCC CTT TGT GAA
HDAC4	NM_053449.1	TGG AAG AGC TGC AGA CAG TG	TCA GCG AGC TGT CCA GTT TC
Cyclophilin A	NM_017101.1	TAT CTG CAC TGC CAA GAC TGA GTG	CTT CTT GCT GGT CTT GCC ATT CC
MYOG	NM_017115.2	GTC CCA ACC CAG GAG ATC ATT T	CCG TTG AGG GGC ATT AAC AA
DACH2	XM_017588282	AGA TTG CAC CAA CGC CAG AA	GGG AGA TCC AGA CGA TTG CTT A
CAMKII	XM_0175900609	TGC CTT TGA ACC CGA AGC AT	GAA CAT CCG TCC AGT AGT TGA T
